# Parents know best: transgenerational predator recognition through parental effects

**DOI:** 10.7717/peerj.9340

**Published:** 2020-06-18

**Authors:** Jennifer A. Atherton, Mark I. McCormick

**Affiliations:** College of Science & Engineering, James Cook University of North Queensland, Townsville, Queensland, Australia; ARC Centre of Excellence for Coral Reef Studies, Townsville, Queensland, Australia

**Keywords:** Alarm odours, Embryos, Olfaction, Parental effects, Predator recognition, Antipredator behaviour

## Abstract

In highly biodiverse systems, such as coral reefs, prey species are faced with predatory threats from numerous species. Recognition of predators can be innate, or learned, and can help increase the chance of survival. Research suggests that parental exposure to increased predatory threats can affect the development, behaviour, and ultimately, success of their offspring. Breeding pairs of damselfish (*Acanthochromis polyacanthus*) were subjected to one of three olfactory and visual treatments (predator, herbivore, or control), and their developing embryos were subsequently exposed to five different chemosensory cues. Offspring of parents assigned to the predator treatment exhibited a mean increase in heart rate two times greater than that of offspring from parents in herbivore or control treatments. This increased reaction to a parentally known predator odour suggests that predator-treated parents passed down relevant threat information to their offspring, via parental effects. This is the first time transgenerational recognition of a specific predator has been confirmed in any species. This phenomenon could influence predator-induced mortality rates and enable populations to adaptively respond to fluctuations in predator composition and environmental changes.

## Introduction

Predation is a major driving force in population and community dynamics (Pettorelli et al., 2011). Antipredator behaviours are often energetically costly and detract from other fitness-promoting activities, like foraging (Werner & Anholt, 1993). Furthermore, the types of predators that pose threats to individuals also change with habitat and life history stage (Lönnstedt & McCormick, 2011). In order to increase their chance of survival, individuals need to be able to recognise predatory threats, and react in a manner that matches the level of risk experienced (Helfman, 1989). Although some species have demonstrated innate recognition of predators (Hawkins, Magurran & Armstrong, 2004), for the majority, learning plays an important role in the identification of relevant threats (Crane & Ferrari, 2013). This learning can occur through direct experience with a predator (e.g., an unsuccessful strike) and also through conditioning events, which can be facilitated using chemical alarm odours in aquatic taxa. These olfactory cues are released when the epidermis of an aquatic organism is damaged, alerting both conspecifics, and closely related heterospecifics (e.g., Mitchell, Cowman & McCormick, 2012; Mitchell et al., 2013), of nearby predatory risks. The temporal coupling of ecologically relevant alarm odours with predator odours, which are passively released, can allow prey to identify the predator as a threat through this common form of Pavlovian conditioning (Suboski, 1990; Ferrari, Wisenden & Chivers, 2010). Some species use olfactory associative learning to further refine innate predator recognition (Berejikian, Tezak & LaRae, 2003). This learned predator information can then be passed on to other individuals within a guild through social learning (Manassa, McCormick & Chivers, 2013). Once learnt, identities of predators can also be generalised to odours from closely related species that may pose a threat (Ferrari & Chivers, 2009). Despite the potential advantages of parents passing on information to their offspring about the identity of relevant predators through non-genetic inheritance, broadly known as parental effects, the extent to which this occurs has seldom been studied.

The parental transfer of information can occur either directly, through inheritance of non-genetic material from one or both parents (i.e., during gametogenesis; Jensen, 2013), or indirectly, as a result of parental behaviour and the care provided to offspring (i.e., during embryogenesis; Bernado, 1996). The parental environment and/or parental attributes can influence the offspring phenotype (in terms of growth and development) and behaviour (Green, 2008; Bennett & Murray, 2014). In addition, environmental cues can be transferred to offspring during gametogenesis, which can consequently alter offspring phenotypes (Ledón-Rettig, Richards & Martin, 2013) and potentially make them more competitive in natal environments (Giesing et al., 2011).

Predator presence can also impact offspring through parental effects. Experimentally exposing breeding pairs to predators can lead offspring to adopt risk averse behaviours (Storm & Lima, 2010; McGhee et al., 2012; Brachetta et al., 2018) as well as influencing their growth and development (Agrawal, Laforsch & Tollrian, 1999; Shine & Downes, 1999; Coslovsky & Richner, 2011a) in a range of taxa. Several potential pathways through which this transfer of information may occur have been suggested. For example, predator presence can induce parental stress, which in turn can alter the hormonal composition of gametes during development and results in changes in offspring quality (McCormick, 1998; Coslovsky et al., 2012). Other research suggests epigenetic mechanisms of transfer, whereby changes in gene expression generate phenotypic differences in offspring (Youngson & Whitelaw, 2008; Mommer & Bell, 2014). Such predator-induced parental effects have been shown to carry-over across all subsequent life stages of the offspring, even into adulthood (Roche, McGhee & Bell, 2012). This phenotypic inheritance can even be detected across multiple generations, affecting factors such as maturation rates and reproductive success (Sheriff, Krebs & Boonstra, 2010; Auld & House, 2015; Walsh et al., 2015). The prevalence of predator-induced parental effects across a wide range of taxa and environments suggests they could confer an adaptive advantage (Mousseau & Fox, 1998; Bestion et al., 2015). However, there are also examples of maladaptive consequences of parental effects in stressful environments, such as increased parasite loading (Coslovsky & Richner, 2011b), decreased antipredator behaviours (McGhee et al., 2012), and metabolic and functional disorders (Schuler & Orrock, 2012) in offspring.

Predator-induced mortality rates in juveniles are exceptionally high in coral reef fishes (Almany & Webster, 2006), as they are for many organisms with complex life cycles (Wilbur, 1980). Recent research has shown that embryonic clownfish (*Amphiprion melanopus*) are able to learn predatory threats through ambient odours (Atherton & McCormick, 2015), and two species of coral reef damselfish responded to alarm odours in a threat-sensitive manner prior to hatching (Atherton & McCormick, 2017). Many coral reef fishes recruit to their natal reefs (Berumen et al., 2012), and identification of known predator odours can influence habitat selection when settling on the reef (Dixson, 2012). Therefore, one would expect that the more parents can prepare their offspring to recognise odours that are relevant to the environment they are likely to settle in, the higher the offspring’s chance of survival. Hence, if parents can impart the ability to identify predators relevant to their natal habitat to their offspring, the larvae that return to these areas are more likely to be able to avoid these at settlement (Vail & McCormick, 2011). Surprisingly, it is currently unknown whether information concerning the identity of specific predator species can be transmitted via parental effects.

Consequently, the aim of our research was to determine if transgenerational predator recognition occurs in a common damselfish on Indo-Pacific reefs, the spiny chromis, *Acanthochromis polyacanthus*. We achieved this by subjecting breeding pairs to one of three olfactory and visual treatments (predator, herbivore, or control), and the offspring produced were then tested for their reactions to one of five olfactory cues (parental predator, novel predator, herbivore, embryo chemical alarm odour, or seawater). Embryo alarm odours were used in the trials to provide a baseline for how embryos respond to a high risk odour, and a novel predator odour was used so we could determine if any reactions to the parental predator odour represented embryos responding to a threat odour in general, or to the transgenerational relay of specific predator information. Here, we show that not only are parents able to convey specific predator information to their offspring, but embryonic damselfish can also innately differentiate between olfactory cues and react according to their level of threat.

## Materials & Methods

### Study species

*Acanthochromis polyacanthus* (Pomacentridae) is an ideal model study species for research into parental effects in coral reef fishes because they can be bred and reared in captivity with minimal mortality, which is partly due to *A. polyacanthus* lacking a pelagic larval stage. Breeding adults form mating pairs and clutches of eggs are laid within small caves in the wild. Eggs are externally fertilized and both sexes guard and maintain the eggs until hatching (Robertson, 1973). Embryogenesis varies in duration from 8–14 days and a brood from a single clutch of eggs can range from 250–550 at hatching, containing juveniles 4.2–4.9 mm standard length (Kavanagh, 2000). In the wild, parents defend their young from reef predators, and parents corral offspring within their natal cave when predators approach. As juveniles get larger they stay together as a shoal until about 3 months old (Kavanagh, 2000). During this time they may learn the identity of predators from shoal mates through mechanisms such as social learning (Manassa & McCormick, 2012).

The model predator species used in treatments and test trials was the coral trout (*Plectropomus leopardus*, Serranidae), a known, sympatric predator of adult and juvenile *A. polyacanthus* (St. John, 1999). The dottyback (*Pseudochromis fuscus*, Pseudochromidae) was used solely as a source of an alternative predator odour in embryo trials and represented the ‘novel predator’ in this experiment. *P. fuscus* is phylogenetically distant from *P. leopardus*, but is another sympatric piscivore of both embryonic and juvenile stage damselfishes (Emslie & Jones, 2001; Feeney et al., 2012). The herbivorous barred rabbitfish (*Siganus doliatus*, Siganidae) was also used in both parental treatments and test trials, and represented a low risk stimulus as a non-threatening coral reef fish species.

### Animal husbandry

All research was completed at the Marine and Aquaculture Research Facilities Unit at James Cook University, Townsville, Australia. Twenty-one adult breeding pairs of *A. polyacanthus* (mean ∼98 mm standard length) were kept in an outdoor, isolated system, with each pair in a 70 L tank. The system contained seawater maintained at 28 ± 1 °C and a salinity of 35 ppt, with a normal light:dark (12.5:11.5 h) diurnal cycle during the summer months. Each tank was well-aerated and contained half of a terracotta pot, which provided both shelter and a substrate on which the breeding pairs could lay clutches of eggs. Each pair was fed pelleted food twice daily and each tank was checked daily for egg clutches; if found, clutches were left with their parents during embryogenesis, but treatments were ceased in tanks containing clutches. At 9 days after fertilisation, all eggs in the clutch were collected by carefully cutting the tissue adhering the eggs to the terracotta pot, and were transferred into a 1 L beaker. The beaker contained water from the parental tank, was well-aerated, and kept in a water-bath in the experimental laboratory to maintain the temperature at that of the parental tank.

### Parental treatments

**Figure 1:**
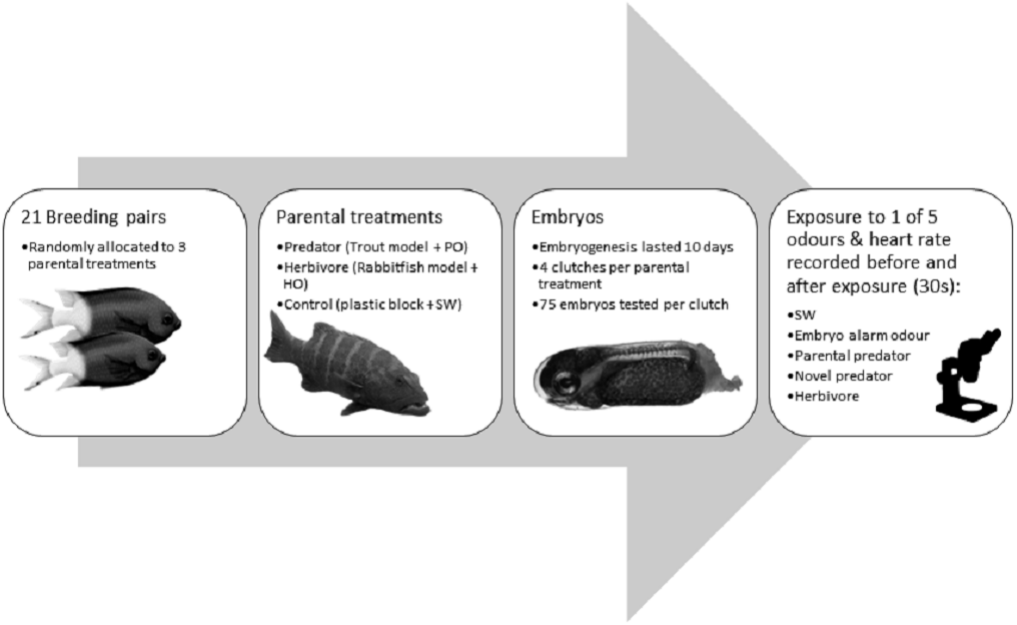
Flow diagram summarising the experimental methdology. Flow diagram of the methods used in the experiment with the coral reef damselfish *Acanthochromis polyacanthus*. Abbreviations: PO, predator odour; HO, herbivore odour; SW, seawater. Embryos were exposed to cues 9 days after fertilization. Image credits: M. McCormick.

Each breeding pair was randomly assigned to one of the three treatments (predator, herbivore, or control), which involved multiple conditioning events, carried out in the morning on a weekly basis, until all test trials had been completed (Fig. 1 for methods summary). Prior to treatments being carried out, all parental tanks were checked for egg clutches. If any clutches were found in parental tanks, treatments were not undertaken for those specific pairs to avoid direct embryo exposure to parental treatment odours. Out of the 21 pairs set up, 3 pairs from each treatment successfully reproduced over the 16 week period that treatments were conducted (September 2014 to January 2015). In order to prevent cross contamination of olfactory cues, the water flow was shut off to each tank, and 8 L of the water in each treatment tank was removed, just prior to treatments, to allow room for the olfactory cues to be added without the risk of overflow into the recirculated system. The predator treatments involved careful introduction of one of two identical fibreglass models of *P. leopardus* (40 cm SL) and 4 L of predator odour, which was slowly added using a funnel tube, so as to reduce the level of human disturbance imposed on experimental fishes. Once every four weeks, to reinforce that the predator odour represented a threat, the 4 L of predator odour was paired with 50 ml of adult *A. polyacanthus* chemical alarm odour (CAO). Research has shown that the co-occurrence of alarm odours with a novel or known odour leads to the secondary odour being categorised as potentially indicative of risk; the more often the pairing, the higher the perceived risk (Ferrari, Sih & Chivers, 2009). During the other three out of four predator treatments within a month, 50 ml of seawater (instead of CAO) was introduced with the predator cue, to ensure consistency of disturbance between treatments. The herbivore treatment involved introducing a fibreglass model of *S. doliatus*, combined with 4 L of herbivore odour and 50 ml of seawater. To act as a disturbance control, a plastic container, 4 L of seawater plus a further 50 ml of seawater were introduced to breeding pairs allocated to control tanks. After 15 min of visual and olfactory cue exposure, around 60% of the water in each tank was siphoned out and the tank was refilled to just below the level of the outflow pipe. The water was left to allow diffusion of all treatment cues for around 4 h before water flow was resumed, and siphoned water was dumped out of the isolated system. In brief, breeding pairs underwent full cue exposure (visual and chemical) for 15 min, followed by 4 h exposure to the substantially diluted chemical cue to allow full degradation of any remaining olfactory stimuli, prior to recommencement of water flow. This procedure ensured that there was no cross-contamination of any olfactory cues among treatment tanks. Research has shown that alarm odours for at least one coral reef fish are degraded by warm water and UV light within 30 min (Chivers et al., 2013).

### Stimulus preparation

#### Treatment odours

Seawater used in the control treatment was obtained from the outdoor parental system to ensure that there was no contamination from other fish odours and that water quality parameters were kept constant. The parental predator odour was obtained from one of three (individual used was changed each week) adult coral trout, *Plectropomus leopardus* (40 cm SL), which were not fed 24 h prior to odour collection to minimise the amount of dietary cues in the water. The predator was kept overnight in 70 L of seawater, and the odour-infused water was then used to treat the predator-assigned breeding pairs. The herbivore odour was produced using the same method, but with the three barred rabbitfish, *Siganus doliatus*, (20 cm SL) as the olfactory cue donor, and the holding tank contained 35 L of seawater. Once a month, the predator odour was combined with an adult *A. polyacanthus* alarm odour, to ensure the breeding pairs were identifying the coral trout as a threat. This alarm odour was created by making ten superficial cuts along each side of an adult (>7 cm SL) *A. polyacanthus*, that had been euthanised by a quick blow to the head, rinsing each side with 50 ml of seawater, using a coarse filter (0.75 mm pore size) to remove any particulate matter. One adult fish was used to make 100 ml of alarm odour, which was enough for two replicates.

#### Trial odours

Embryo reactions were tested, using one of five different olfactory cues, which included: seawater control (SW), embryo chemical alarm odour (CAO), parental predator (coral trout, *P. leopardus*), novel predator (dottyback, *P. fuscus*), or herbivore (rabbitfish, *S. doliatus*). The *A. polyacanthus* embryo CAO was made by crushing five embryos in a petri dish and mixing this with 5 ml of seawater. The resultant solution was filtered through filter paper to remove particulate matter, leaving the odour infused seawater; 1 ml of this olfactory cue was used in each test trial. Odours for the parentally taught predator and herbivore trials were collected at the same time as the water for the weekly odour conditioning (above ‘*Treatment odours*’) and 2 ml aliquots were placed in liquid nitrogen for a maximum of 2 weeks, to be defrosted and used when trials were carried out (Table S1). The dottyback (10 cm SL) was placed in 9 L seawater overnight to create the novel predator odour, and the resultant olfactory cue was also frozen in 2 ml aliquots. Again, the dottyback was starved 24 h prior to olfactory cue collection to minimise the presence of digested alarm odours in the trial odours.

### Embryo test trials

Heart rate was used as the measured behavioural proxy because it is easily visible using a dissecting microscope and research has shown that predator presence can induce not just behavioural changes, but also concurrent changes in heart rate (Höjesjö, Johnsson & Axelsson, 1999). A total of 75 embryos (day 9 post-fertilization) were tested from each clutch; 15 embryos for each of the five odours. A single embryo was placed in 10 ml of seawater, sourced from the same temperature controlled system, under a dissecting microscope with a cold light, and allowed 2 min to acclimatise. The reaction elicited by the introduction of a test odour was calculated by visually recording the embryo’s heart rate for 30 s, carefully injecting 1 ml of one of the five olfactory cues into the seawater, and then recording the heart rate for a further 30 s. This procedure was repeated using four separate clutches, from three breeding pairs (two pairs produced one clutch each and one pair produced two clutches), for each parental treatment. Consequently, 60 embryos from four clutches were tested against each of the five trial odours, for each of the three parental treatments (900 embryos tested in total). Measurement of 30 random eggs per treatment found that there was no difference in the maximum length of eggs among treatments (ANOVA, *F*_2,6_ = 2.12, *P* = 0.2).

### Statistical analyses

#### Baseline heart rates

A nested ANOVA model was conducted to assess if actual embryo heart rates, prior to the introduction of trial odours, differed across parental treatments. This two-factor model tested parental treatment (fixed) and clutch (random and nested in parental treatment). Residual analysis showed that the raw initial heart rate data met the assumptions of ANOVA.

#### Changes in heart rates

A three-factor partially nested ANOVA was undertaken to determine whether the change in embryo heart rate was affected by: parental treatment (fixed: predator, herbivore, seawater); trial odour (fixed: parental predator, novel predator, herbivore, embryo alarm odour, seawater); and clutch (random and nested in parental treatment: four clutches). Change in heart rate was calculated as a percentage, by deducting the heart rate recorded pre-stimulus from the heart rate post stimulus, and multiplying by 100. To determine whether having two rather than one clutch of eggs from one parent in each of the treatments unduly biased the outcome of the experiment, a preliminary analysis was undertaken that did not include the second clutch from these breeding pairs. This analysis on the reduced dataset showed exactly the same patterns of significance and very similar effect sizes to the complete dataset, suggesting that the addition of an extra clutch from some pairs did not bias the results. Effect sizes are given as partial-eta-squared, which represents the proportion of the total variance in a dependent variable that is associated with the membership of different groups. Tukey’s HSD post-hoc tests were used to determine the nature of any significant differences found by ANOVA. The raw change in heart rate data also met the assumptions of ANOVA. Statistical analyses were conducted using Statistica (version 13).

### Ethical approval

All procedures were approved by the James Cook University Animal Ethics Committee under the permit A1871, and all guidelines for the care and use of animals were followed as per this permit.

## Results

### Baseline heart rates

**Figure 2:**
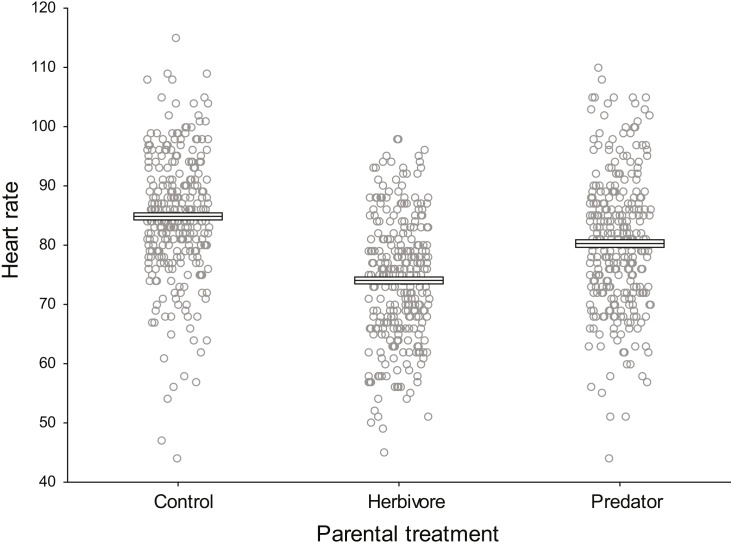
Baseline damselfish embryo heart rates prior to commencement of experimental test trials. Mean baseline embryo heart rates for each parental treatment prior to the introduction of trial odours (*N* = 300 per parental treatment). Mean and standard errors are shown as boxes with a central line, while all data for each treatment are plotted as circles.

Baseline embryo heart rates (beats 30 s^−1^ prior to odour introduction) did not differ significantly among parental treatments (*F*_2,9_ = 2.33, *P* = 0.154, *η*_p_^2^ = 0.34, Fig. 2). Clutch nested within parental treatment was significant (*F*_9,888_ = 51.18, *P* <0.0001, *η*_p_^2^ = 0.34) indicating there were differences in the heart rate of embryos among clutches within a parental treatment.

### Reactions to trial cues based on parental treatment

**Figure 3:**
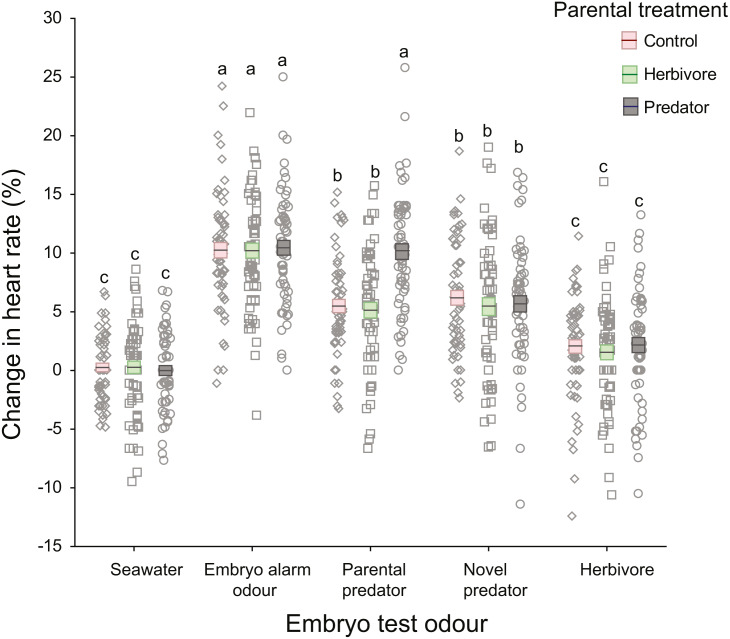
Changes in heart rates of embryonic damselfish from three parental treatment groups, in response to five chemosensory test cues. Mean change in embryo heart rate (%) after introduction of one of the five test odours. Mean and standard errors are shown as boxes with a central line, while all data for each treatment are plotted as circles. Red boxes represent the upper and lower standard errors for embryos from the parental control treatment, while green and grey represent herbivore- and predator-parental treatments respectively (*N* = 60, representing 15 embryos from each of four clutches per parental treatment). Lowercase letters above the data represent Tukey’s HSD groupings of means.

There was a significant interaction between parental treatment and embryo trial odour (Table 1; Fig. 3). This interaction was driven by embryos from the parental-predator treatment responding differently to the introduction of parental predator odour (coral trout) compared to embryos from the other parental treatments (Fig. 3). Following introduction of the parental predator odour, offspring from the predator treated parents showed an increase in heart rate (+10.13%) that was almost twice that of embryos from the herbivore and seawater control treated parents (+5.14%, +5.49%, respectively; Tukey’s HSD: *P* < 0.001 for both comparisons; Fig. 3). This contrasted with the heart rate changes induced by the seawater, embryo alarm odour, novel predator, and herbivore trial odours that did not differ among the three parental treatments (Tukey’s HSD: *P* = 1.00 for each of the four aforementioned odours, when comparing across parental treatments; Fig. 3). There was a significant difference between clutches (nested within parental treatments) in changes in heart rate, but this was consistent across test cues (non-significant interaction, *p* = 0.17, Table 1). This suggests that the sensitivity of the embryos was dependent upon clutch identity, which is likely to be parentally driven. The effect sizes were high for all significant terms (*η*_p_^2^ > 0.5), with the highest being for the effects of the test cue and their interaction with parental treatments (*η*_p_^2^ = 0.99 and 0.84, respectively; Table 1).

**Table 1:** Comparing the reactions of embryonic damselfish, whose parents experienced different levels of predatory treatments, to varying levels of threat odours. Comparison of the mean changes in heart rates of embryonic *Acanthochromis polyacanthus*, that were exposed to one of five chemosensory cues (parental predator, novel predator, herbivore, embryo alarm odour, seawater), and whose parents had been exposed to one of three threat treatments (predator, herbivore, seawater) of four egg clutches.

Effect	*df*	MS	*F*	*P*	*Effect size*
Parental treatment (P)	2	108.46	5.82	0.024	0.56
Embryo cue (E)	4	2,935.34	764.06	<0.0001	0.99
P × E	8	93.83	24.42	<0.0001	0.84
Clutch (Parental treatment) C(P)	9	18.62	4.85	0.0003	0.55
E × C(P)	24	3.84	0.17	1.0000	0.01
Residual	840	23.11			

### Reactions to cues irrespective of parental treatment

On introduction of an embryo chemical alarm odour (CAO), embryos from all three parental treatments responded with a similar increase in heart rate (mean = +10.31%), which differed significantly from the mean increases in heart rate elicited by the seawater, novel predator and herbivore odours (Tukey’s HSD: *P* <0.001 for all three comparisons; Fig. 3). Similarly, reaction of the embryos to the herbivore odour did not differ significantly across the three parental treatments, but the mean increase in heart rate of +1.92% differed significantly from those of all the other test odours (Tukey’s HSD: *P* <0.005), except for the seawater control (Fig. 3). The introduction of a novel predator odour induced an increase in heart rate that was similar across parental treatments (mean = +5.59%; Tukey’s HSD: *P* = 1.00) and was the same as that for embryos exposed to coral trout odour whose parents had been conditioned with either herbivore or control conditions (Fig. 3).

## Discussion

Our findings suggest that specific predator information may be being passed across generations, through non-genetic parental effects. Introduction of a parentally-known predator odour to the vicinity of the embryos induced an almost two-fold increase in heart rate for the offspring from the predator-treated parents, compared to the offspring from the herbivore and control parental treatments. As *Acanthochromis polyacanthus* brood their young for up to three months after hatching (Kavanagh, 2000), the predators experienced by parents are likely to mirror those present in their offspring’s environment. The ability of parents to forewarn their offspring of predatory risk has also resulted in a more frequent occurrence of general antipredator behaviours in: three-spined sticklebacks (*Gasterosteus aculeatus*; Giesing et al., 2011), fall field crickets (*Gryllus pennslyvanicus*; Storm & Lima, 2010 and the Tussock skink (*Pseudemoia pagenstecheri*; Shine & Downes, 1999). Additionally, research has demonstrated that parents in high risk environments can increase progeny survival by producing offspring with desirable morphological traits, such as faster growth rates (Besson et al., 2014), and induced defences (Tollrian, 1995). Combined, these phenotypically plastic traits suggest that some parents can gear their offspring to the challenges they are likely to face during early life stages. Still, to our knowledge, our study is the first to demonstrate that offspring can differentiate between predator odours by showing an increased reaction to a specific predator experienced by their parents, and not just a transgenerational response to a risky environment.

In this study, we used changes in heart rates to determine if damselfish embryos recognised and/or reacted to chemosensory information. If the embryos recognised a cue to be potentially detrimental or beneficial, we expected to see a change in their heart rate. To reinforce that the stimuli represent a significant threat for the parents, we gave the parents exposed to the coral trout stimuli a conspecific alarm odour once per month with the predator stimuli (i.e., sight and smell of a coral trout). This co-occurrence of an alarm odour with another stimulus is a fundamental method whereby aquatic organisms learn threats and update information about risk (Ferrari, Wisenden & Chivers, 2010). It is possible that this alarm odour alone may have caused parents to prime offspring to better survive in a high risk environment, without any information specific to the coral trout predator. The addition of ‘alarm odour alone’, and ‘coral trout odour alone’ treatments would have been beneficial to determining the transgenerational importance of alarm odours in the present experiment. It has been recently shown that exposure to alarm odours can lead to neophobia, where the recipients demonstrate marked antipredator responses to any novel stimuli (McCormick et al., 2017), but this mechanism of behavioural alteration has yet to be shown to function across generations. The results of the present experiment suggest that the effect is not simply a transgenerational forewarning of a stressful natal environment, but rather information specific to a particular predator type. We found that offspring from parents exposed to a specific predator (coral trout in conjunction with an alarm odour), prior to spawning responded to the olfactory cues of that specific predator with a greater intensity compared to controls or a novel odour (dottyback or herbivore). Given the small size of the juvenile *Acanthochromis* at hatching, the dottyback represents a more relevant predator to the juveniles than a coral trout, suggesting that parental exposure paired with an alarm odour has upgraded the relevance of these odours for their offspring, in keeping with the risk-allocation expectations. This parentally endowed information may potentially be generalized to all members of the genus of coral trout (*Plectropomas*) or elicit a graded response in relation to the phylogenetic closeness of the cue to the conditioning odour, as has been shown for odour learning in fishes generally (e.g., Ferrari et al., 2007; Mitchell et al., 2013). The generality of the information parentally transferred to their offspring is presently unknown, but it appears that they can at least pass on information about the identity of specific species that represents a known threat.

While our results show a clear distinction among the reactions of embryos to different olfactory cues, the potential adaptive significance of embryos showing tachycardic responses to threat odours is unknown. We also do not know the temporal duration of the elevated heart rates found. Research into both aquatic and terrestrial prey species has shown that increases in heart rates often accompany antipredator behaviours and denote predator recognition (Smith & Johnson, 1984; Johnsson, Höjesjö & Fleming, 2001). Ydenberg & Dill (1986) also suggested that neurophysiological responses (e.g., changes in heart rate) can provide insight into predator awareness, prior to any observable flight behaviour. Although existence of embryonic tachycardic responses to threat odours could imply some form of selective survival benefit (Oulton, Haviland & Brown, 2013; Atherton & McCormick, 2015; Atherton & McCormick, 2017), failing to couple this reaction with a predator avoidance response (e.g., premature hatching, as seen in Cohen, Seid & Warkentin (2016), but not in this study) would still result in increased energy expenditure (Palacios et al., 2016). Consequently, embryos may consume their yolk reserves at a greater rate, which may lead to smaller larvae that have lower survival upon hatching (Blaxter & Hempel, 1963; McCormick & Nechaev, 2002). Interestingly, in the longer term organisms may acclimate to high threat conditions. A study on fishes found that although exposure to a predatory threat induced tachycardia, prolonged exposure resulted in a reduction of overall resting heart rate and activity levels (Holopainen et al., 1997). Steiner & Van Buskirk (2009) found a similar trend in tadpoles, but with oxygen consumption rather than heart rate. In both examples, this long term reduction in metabolic activity in risky environments allowed for more energy to be allocated to growth, which should be beneficial. Further research is warranted to determine the longer term consequences of the tachycardia demonstrated in the current study for organism fitness.

While parental exposure to predators can lead to beneficial offspring characteristics, there is also evidence to suggest that parental predator exposure may have maladaptive consequences for offspring fitness (McGhee et al., 2012). This is likely to be a result of predator presence increasing the concentration of stress hormones, such as cortisol, which can subsequently transfer into the eggs of gravid mothers (reviewed in Green, 2008, see McCormick, 2009). Coslovsky & Richner (2012) suggested that if there is a mismatch between the maternal environment and that of the resultant offspring, offspring fitness may suffer as a result of being geared to suit the wrong environment. In the context of the present study, the ability of parents to convey specific predator information to their offspring may provide them with a means for early recognition and escape from predators. Yet, if the conveyed predator information is not pertinent to their offspring’s life stage (i.e., due to gape limitations in predators; St. John, 1999), the offspring could incur an energetic cost by reacting to a non-relevant predator (Helfman, 1989). However, it is possible that any maladaptive effects caused by maternal stress hormones could be overridden by an individual’s own experiences (Stratmann & Taborsky, 2014), current environmental conditions (Donelson, Munday & McCormick, 2009), or by demonstrating flexibility in growth later in life (Gagliano & McCormick, 2007). For example, Feng and colleagues (2015) demonstrated that by becoming more reliant on social cues, offspring can overcome the reduced learning capabilities caused by maternal stress. This could be particularly pertinent in complex ecosystems, such as coral reefs, where social learning is likely to be very important (Manassa & McCormick, 2013).

A number of mechanisms have been suggested as the means of transgenerational information transfer; namely, hormonal (McCormick, 1998; Groothuis & Schwabl, 2008; Coslovsky & Richner, 2011a; Ensminger et al., 2018) and epigenetic (Youngson & Whitelaw, 2008; Mommer & Bell, 2014), but this is still a relatively new and largely speculative field of research. In the current study, we also have the possibility of some form of information transfer to the embryos from the parents as they tended the eggs (remember that embryos were left with the parents until day 9, during which time the parents were no longer conditioned). If this was the case, then any parental influence would likely be from products from the urogenital system (e.g., cortisol, testosterone) or through the egg-tending regime (e.g., Neff & Knapp, 2009) and one may expect this to lead to a general neophobia in embryos. This seems unlikely due to the specificity of the transgenerational predator recognition observed in this study. Furthermore, while efforts were made to ensure embryos were not directly exposed to the parental treatments, it is plausible that olfactory cues could have been received during gametogenesis (while the eggs were still developing in the mother). These environmental cues could alter the development and behaviour of resultant offspring (Ledón-Rettig, Richards & Martin, 2013), though this has yet to be demonstrated for fishes.

Our findings also demonstrate that damselfish have innate recognition of predatory threats, indicated by the increase in heart rate induced by a novel predator odour (coral trout and/or dottyback). In this context, we refer to the ‘innate recognition’ as a reaction observed in an embryo in response to an olfactory stimulus, which the embryo itself has not previously experienced. While neophobic responses to threat odours are present in some species and situations (Brown et al., 2013; Meuthen et al., 2016; McCormick et al., 2017), the increases in heart rate seen in our study is unlikely to be a result of neophobia. This is because the embryos showed a significantly greater reaction on introduction of an unknown predator odour compared to the herbivore odour - both of which were ‘novel’ olfactory cues. The seemingly innate ability of prey to recognise a predatory threat by smell could also be a result of the recognition of a common digestive product released by piscivores after consuming similar prey species (Mirza & Chivers, 2003; Schoeppner & Relyea, 2005). Innate predator recognition has been identified in other species (Hawkins, Magurran & Armstrong, 2004; Oulton, Haviland & Brown, 2013), but this knowledge is often further enhanced through associative learning (Epp & Gabor, 2008; Atherton & McCormick, 2015), or as our findings also suggest, upregulated by parental effects. Using a combination of mechanisms for recognising predatory threats may be important in life stages and environments with a high risk of predation. As such, when considering the impact predators have on offspring success and population dynamics, a combination of factors, namely parental effects, offspring’s own experiences and phenotypes, and genetics, all need to be taken into account (Stratmann & Taborsky, 2014; Donelan & Trussell, 2018; Tóth & Hettyey, 2018).

## Conclusions

Our findings suggest that not only are parents able to convey species-specific predator information to their offspring, but as embryos, offspring also have astute olfactory capabilities with which they can gather additional information regarding local threats before hatching. However, further research is required to identify the long term consequences predator-induced parental effects have on offspring development, behaviour, and survival in later life stages (Chaby, 2016), and determine the mechanism for transfer of predator information in this coral reef damselfish. Olfactory recognition of predatory threats in embryos could provide a potentially adaptive mechanism for survival, but it seems that post-hatching plasticity may be the key to either overwriting any potential negative consequences of predator-induced parental effects or building on any relevant predatory information transferred.

##  Supplemental Information

10.7717/peerj.9340/supp-1 Supplemental Information 1 Pilot trial assessing the viability of using frozen chemosensory cues instead of freshly collected fish odours A two-factor ANOVA comparing the change in heart rate induced by the type of dottyback (*Pseudochromis fuscus*) cue used (fresh or frozen), and the clutch from which the *Acanthochromis polyacanthu* s embryos were sourced.

## Additional Information and Declarations

### Data Availability

The following information was supplied regarding data availability: Data are available at JCU’s Tropical Data Hub: Atherton, J. (2019): Transgenerational predator recognition through parental effects. James Cook University. (dataset). DOI: 10.25903/5caebe17a0ddc.
