# Supplementary material for: Parents know best: transgenerational predator recognition through parental effects
Source: PeerJ. 2020 Jun 18;8:e9340. doi: 10.7717/peerj.9340 (PMC7306219; doi:10.7717/peerj.9340)
Supplement: Supplemental Information 1 — A two-factor ANOVA comparing the change in heart rate induced by the type of dottyback (Pseudochromis fuscus) cue used (fresh or frozen), and the clutch from which the Acanthochromis polyacanthu s embryos were sourced. [file peerj-08-9340-s001.docx]

**Pilot trial**

A pilot study was undertaken to determine whether it is possible to use fish odours that had been frozen in liquid nitrogen as a viable replacement for freshly collected odours, using the example of the dottyback, *Pseudochromis fuscus*. The fresh dottyback odour induced a 5.81% increase in heart rate, compared to the 5.51% increase caused by the frozen and defrosted odour; a two-factor ANOVA showed there to be no significant difference between the two (Table S1). Freezing and storing odours prior to test trials reduced the amount of handling stress placed on all three species of fishes from which trial and test odours were obtained.

**Table S1:** A two-factor ANOVA comparing the change in heart rate induced by the type of dottyback (*Pseudochromis fuscus*) cue used (fresh or frozen), and the clutch from which the *Acanthochromis polyacanthus* embryos were sourced.

| Effect | *df* | MS | *F* | *P* |
| --- | --- | --- | --- | --- |
| Pair | 2 | 16.55 | 0.721 | 0.4890 |
| Cue type | 1 | 2.03 | 0.088 | 0.7670 |
| Pair x Cue type | 2 | 0.55 | 0.024 | 0.9763 |
| Residual | 84 | 22.94 |  |  |
